# Contactin 1 modulates pegylated arginase resistance in small cell lung cancer through induction of epithelial–mesenchymal transition

**DOI:** 10.1038/s41598-019-48476-8

**Published:** 2019-08-19

**Authors:** Shi Xu, Sze-Kwan Lam, Paul Ning-Man Cheng, James Chung-Man Ho

**Affiliations:** 1 Division of Respiratory Medicine, Department of Medicine, The University of Hong Kong, Queen Mary Hospital, Pokfulam, Hong Kong SAR China; 2 Department of Burn and Plastic Surgery, Shenzhen Longhua District Central Hospital, Affiliated Central Hospital of Shenzhen Longhua District, Guangdong Medical University, Shenzhen, Guangdong China; 3 Bio-cancer Treatment International, 511-513, Bioinformatics Building, Hong Kong Science Park, Tai Po, Hong Kong SAR China

**Keywords:** Small-cell lung cancer, Cancer therapeutic resistance

## Abstract

Drug resistance is a major hurdle in the treatment of small cell lung cancer (SCLC). Previously we demonstrated the potential anticancer effect of pegylated arginase BCT-100 in SCLC cell lines and xenograft models. To facilitate future clinical application of BCT-100 in SCLC treatment, we elucidated the potential mechanisms that underlie acquired drug resistance to BCT-100. H446 and H526 SCLC cells were serially cultured in stepwise increasing concentrations of BCT-100 until stable BCT-100-resistant cell lines emerged (H446-BR and H526-BR). Compared with parent cells, H446-BR and H526-BR displayed stronger migration ability, anoikis resistance and EMT progression. Gene chip assay was employed to select three potential targets (CDH17, CNTN-1 and IGF2BP1). Silencing CNTN-1 rather than CDH17 or IGF2BP1 in H446-BR and H526-BR cells re-sensitized resistant cells to BCT-100 treatment and attenuated the epithelial–mesenchymal transition (EMT) phenotype. The AKT signaling pathway was activated in H446-BR and H526-BR cells accompanied by EMT progression, and AKT inhibitor LY294002 reversed the EMT progression in resistant cells.

## Introduction

Small cell lung cancer (SCLC) is an extremely malignant cancer that poses a great health threat to humans worldwide. Although patients with SCLC have an initially favorable response to chemotherapeutic regimens, most experience relapse with consequent more intractable disease^1^. The cornerstone of treatment for SCLC remains etoposide and cisplatin as first line therapy and topotecan as second line with modest clinical benefits. It is imperative to design novel therapeutic agents that can provide more options and improve the poor outcomes.

BCT-100, a pegylated recombinant human arginase 1, exerts its effect by degrading arginine to ornithine, leading to arginine depletion in the tumor microenvironment^2^. BCT-100 is a potential effective therapeutic agent for tumors that cannot synthesize arginine independently and that were previously considered arginine auxotrophic cancers. These arginine auxotrophic tumor cells lack either argininosuccinate synthase 1 (ASS1) or ornithine carbamoyltransferase (OTC) expression, and thus interrupt the normal urea cycle. The anticancer effect of BCT-100 has been demonstrated in various cancers including human hepatocellular carcinoma (HCC)^3^, melanoma^4^, malignant pleural mesothelioma^5^ and leukemia^6^. In our previous study, some SCLC cell lines also lost the ability to generate arginine endogenously, and BCT-100 exhibited its anticancer effect through induction of oxidative stress and cell cycle arrest in SCLC^7^. To address potential problems with the future clinical application of BCT-100 in SCLC treatment, it is prudent to elucidate the mechanism that underlies acquired drug resistance to BCT-100.

Epithelial-mesenchymal transition (EMT) was initially identified in developing embryos and is a classic example of cellular plasticity in embryonic development as well as in cancer progression^8^. The concept of EMT in cancer research is that tumor cells exhibit an obvious down-regulation of epithelial characteristics, loss of epithelial cell polarity and reduced intercellular adhesion. At the same time, tumor cells acquire mesenchymal stem cell-like properties that include enhanced migratory and invasive abilities. EMT plays essential roles in many biological processes including wound healing, tissue fibrosis, tumor migration and embryo development^9–11^. There is growing evidence that EMT progression is associated with drug resistance in various types of cancer cell^12–14^. Generally, drug resistant cancer cells with EMT progression are characterized by an enhanced ability for cell migration, acquisition of a cancer stem cell-like phenotype and anoikis resistance^15^. Additionally, it has been well reported that EMT progression is closely linked to activation of the AKT signaling pathway, and that this explains the chemotherapeutic drug resistance of several cancers including lung cancer^16^, breast cancer^17^, ovarian cancer^18^ and leukemia^19^. Nonetheless the role of EMT in acquired resistance to pegylated arginase in SCLC remains unclear.

Cadherin-17 (CDH17) belongs to 7D-cadherin superfamily and has important role in intercellular adhesion^20^. It has been reported that CDH17 was overexpressed in gastric cancer^21^, human hepatocellular carcinoma^22^ and colorectal cancer^23^ and was associated with cell proliferation, metastasis and poor prognosis. Silencing CDH17 in gastric cancer cells inhibited cell proliferation and invasion, following NF-κB signaling pathway inactivation^21^. However, the function of CDH17 in multidrug resistance still remains unclear.

Insulin-like growth factor 2 mRNA-binding protein 1 (IGF2BP1) is a highly conserved protein in IGF2BP family, which can interact with RNA and regulate the fate of transcript target^24^. As an oncofetal protein, IGF2BP1 is highly expressed during embryogenesis but negotiable levels in normal human tissues. However, accumulating evidence has showed that IGF2BP1 was re-expressed in various malignant tumors including HCC, melanoma and rhabdomyosarcomas^24–26^. It modulates the drug resistance in rhabdomyosarcomas via mediating cellular inhibitor of apoptosis 1 (cIAP1), which is an essential factor to promote tumor cell survival^26^. Besides, IGF2BP1 is a biofunctional target of miRNA and responsible for suppression on tumor growth and metastasis in cervical cancer^27^. Thus, IGF2BP1 is an attractive target for anti-cancer and anti-drug resistance therapy in clinical practice.

Contactin 1 (CNTN-1) is an adhesion molecule that belongs to the immunoglobulin (Ig) superfamily, and plays an essential role in nervous system development^28,29^. Accumulating evidence has revealed that CNTN-1 is involved in carcinogenesis and cancer progression^30,31^. For example, overexpression of CNTN-1 promotes cell proliferation, colony formation and migration in breast cancer^32^. And inhibition of CNTN-1 in lung adenocarcinoma has been shown to abolish tumor metastasis and prolong survival in a xenograft model^33^. Therefore, CNTN-1 is a promising potential target for cancer therapy.

In this study, we aimed to investigate the mechanisms of acquired resistance to pegylated arginase in SCLC. Acquired resistance models using H446 and H526 cells exposed to increasing concentrations of BCT-100 for a prolonged period were utilized. The BCT-100-resistant (BR) cell lines demonstrated EMT properties which were partially related to up-regulation of CNTN-1. Silencing of CNTN-1 re-sensitized BR cells to BCT-100 treatment indicating that suppression of CNTN-1 might reverse BCT-100 resistance in SCLC patients.

## Results

### H446-BR and H526-BR cells displayed multidrug resistance to BCT-100, cisplatin and etoposide

**Figure 1:**
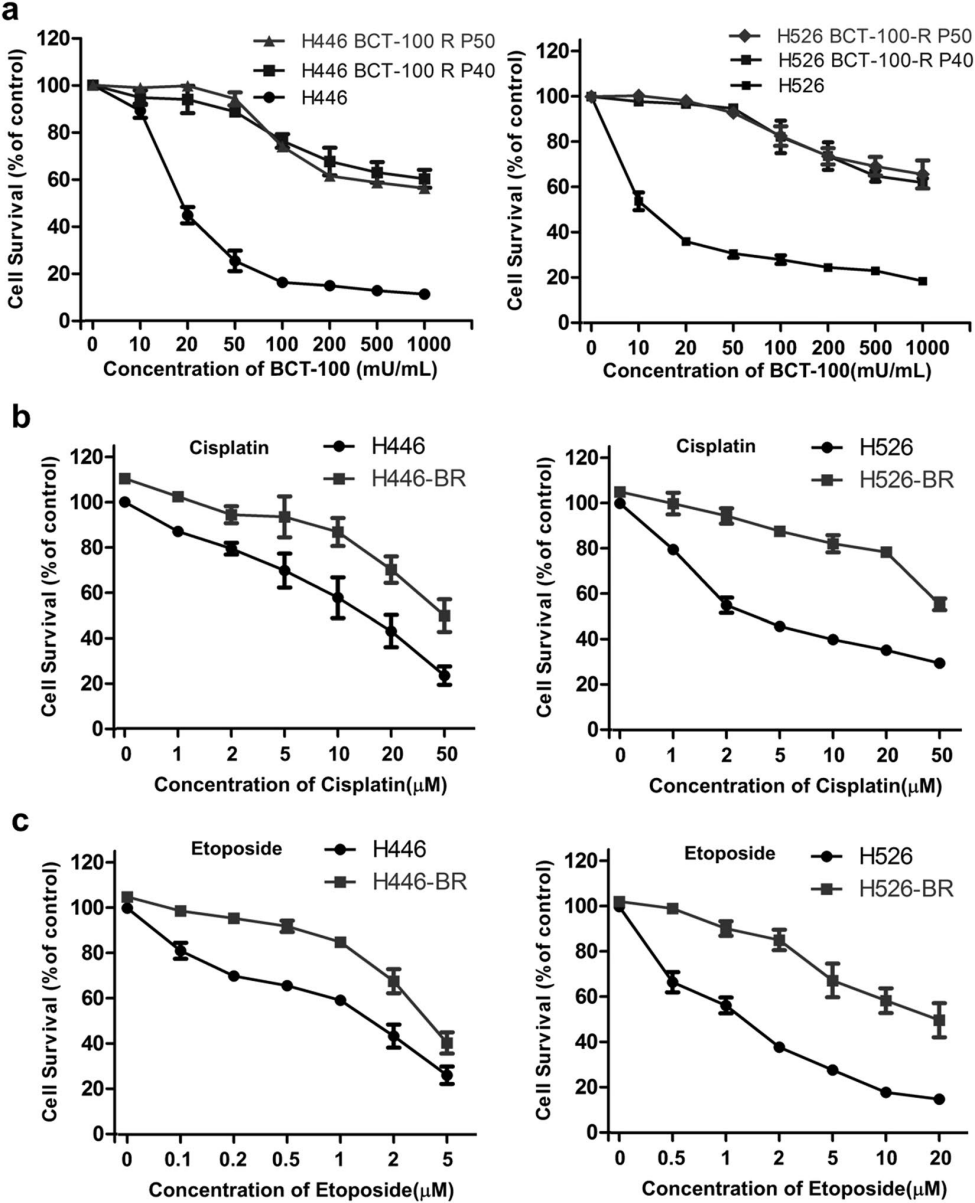
H446-BR and H526-BR cells displayed multidrug resistance to BCT-100, cisplatin and etoposide. (**a**) Cells were exposed to increasing concentrations of BCT-100 ranging from 10 to 1,000 mU/mL, and treatment duration was 3 days. The survival rate plateaued at the 40^th^- passage in H446 and H526 cells. (**b**,**c**) H446-BR and H526-BR cells were more resistant to cisplatin and etoposide compared with parent cells.

H446 and H526 are two cell lines with the ability to develop BCT-100-resistance. After at least 6 months of serial cell culturing with gradually increasing concentrations of BCT-100, selected H446 and H626 cells were able to tolerate exposure to BCT-100 and the cell survival curves reached a plateau after the 40^th ^passage (Fig. 1a). In addition, H446-BR and H526-BR cells were resistant to cisplatin and etoposide compared with their parent cells (Fig. 1b,c). The acquired resistant mechanisms to cisplatin and etoposide remained to be elucidated.

### H446-BR and H526-BR cells displayed an EMT phenotype with aggressive migration ability

**Figure 2:**
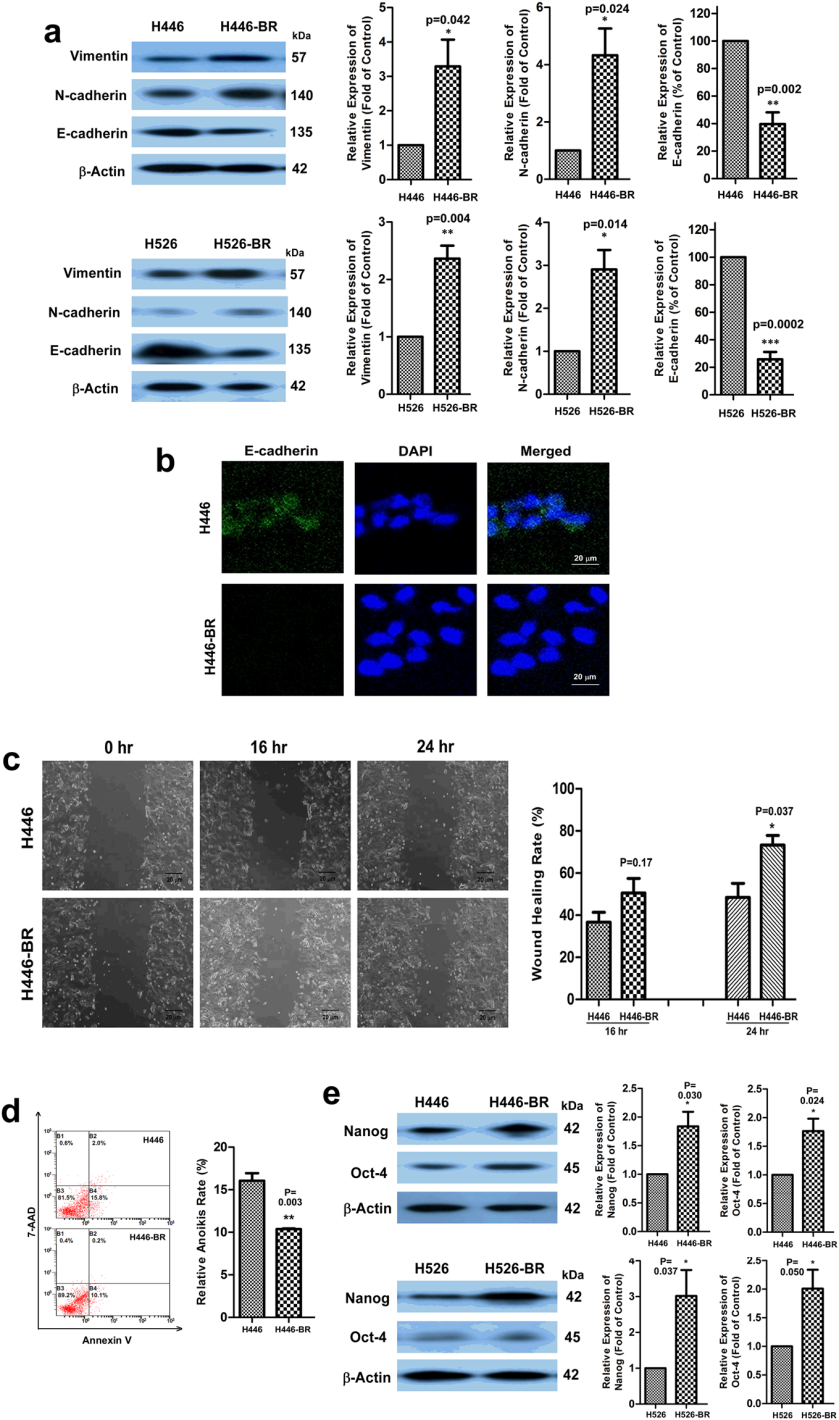
H446-BR and H526-BR cells displayed an EMT phenotype with aggressive migration ability. (**a**) Vimentin and N-cadherin were up-regulated and E-cadherin was down-regulated in BCT-100 resistant cells. (**b**) E-cadherin signal was observed in H466 parent cells but not H446-BR cells by immunofluorescence staining. (**c**) The migration ability of H446-BR cells was higher than that of parent cells. (**d**) Flow cytometry results showing that H446-BR cells had a lower percentage of anoikis cells than H446 cells. (**e**) Expression of Oct-4 and Nanog was higher in BCT-100 resistant cells.

EMT progression was observed in H446-BR and H526-BR cells. The mesenchymal biomarkers N-cadherin and vimentin were elevated in BCT-100-resistant cells, while the epithelial biomarker (E-cadherin) was decreased (Fig. 2a). Immunofluorescence assay also showed that E-cadherin was decreased in H446-BR cells (Fig. 2b). The scratch wound healing assay has been widely used for adherent cells (H446 and H446-BR) to evaluate cell migration ability. Compared with that of H446 cells, the migration ability of H446-BR cells was higher. The wound healing rate increased from 47.5 ± 6.6% (H446) to 70.0 ± 5.9% (H446-BR) in 24 hr (Fig. 2c). The anoikis resistance experiment indicated that the cellular aggregates in H446-BR cells were much larger than those of the parent cells (Supplementary Fig. S1). Flow cytometry analysis of suspended cells showed that H446 cells had a higher percentage (17.8%) of anoikis cells than H446-BR cells (10.3%) (Fig. 2d). Moreover, the pluripotent stem cell biomarkers (oct-4 and nanog) were up-regulated in H446-BR and H526-BR cells (Fig. 2e). However, we did not investigate the presence of pluripotent stem cell in stable cell lines.

### CDH17, CNTN-1 and IGF2BP1 were up-regulated in H446-BR and H526-BR cells

**Figure 3:**
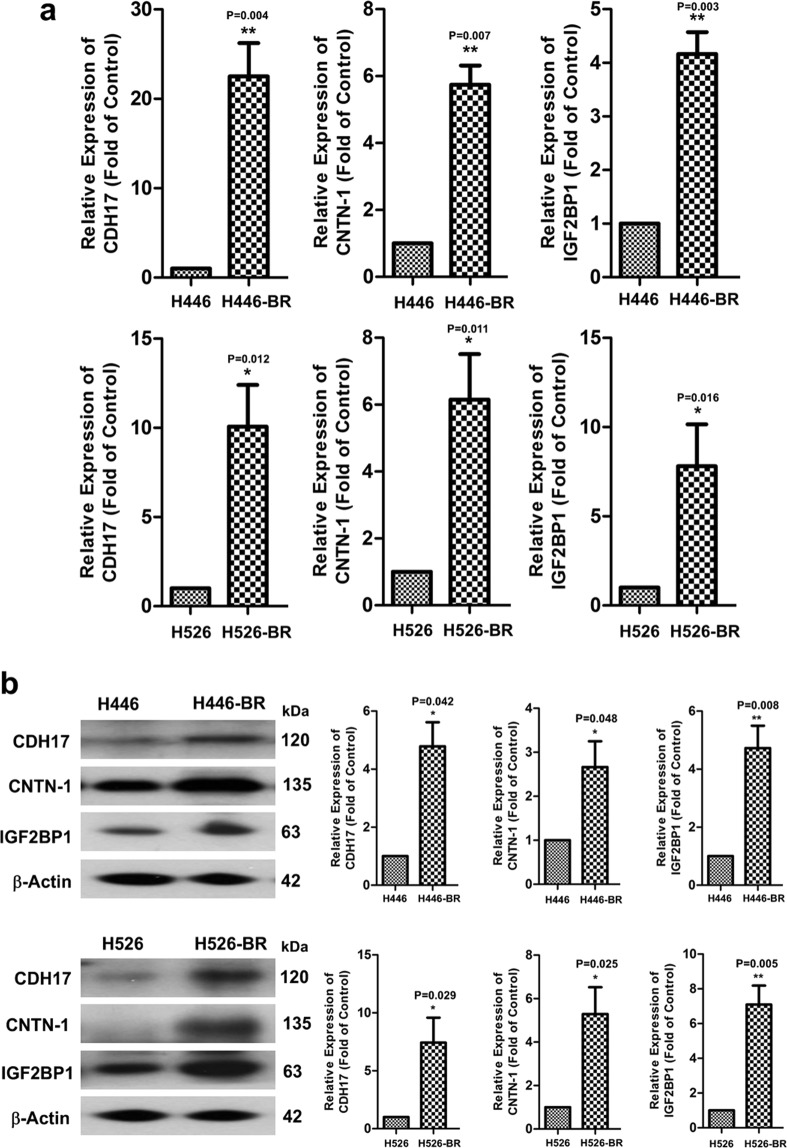
CDH17, CNTN-1 and IGF2BP1 were up-regulated in 446-BR and H526-BR cells. Both mRNA (**a**) and protein (**b**) expression of these three targets were up-regulated in resistant cell lines.

Affymetrix Human Transcriptome Array (HTA) 2.0 was used to compare the gene changes between BCT-100-resistant cells and parent cells. A total of 74654 candidate genes were covered in this assay. Gene alterations over three-fold were selected: 1310 and 129 candidate genes in H446 and H526 cells respectively, although there were 38 common targets among the H446-BR and H526-BR selected genes (Supplementary Table S1). A review of the literature revealed that CDH17, CNTN-1 and IGF2BP1 were associated with tumor progression and drug resistance and were identified as potential candidates for further analysis. Both the gene and protein levels of these targets were increased in H446-BR and H526-BR cells (Fig. 3a,b).

### Knockdown of CDH17 and IGF2BP1 had very little influence on BCT-100 resistance and EMT phenotype

**Figure 4:**
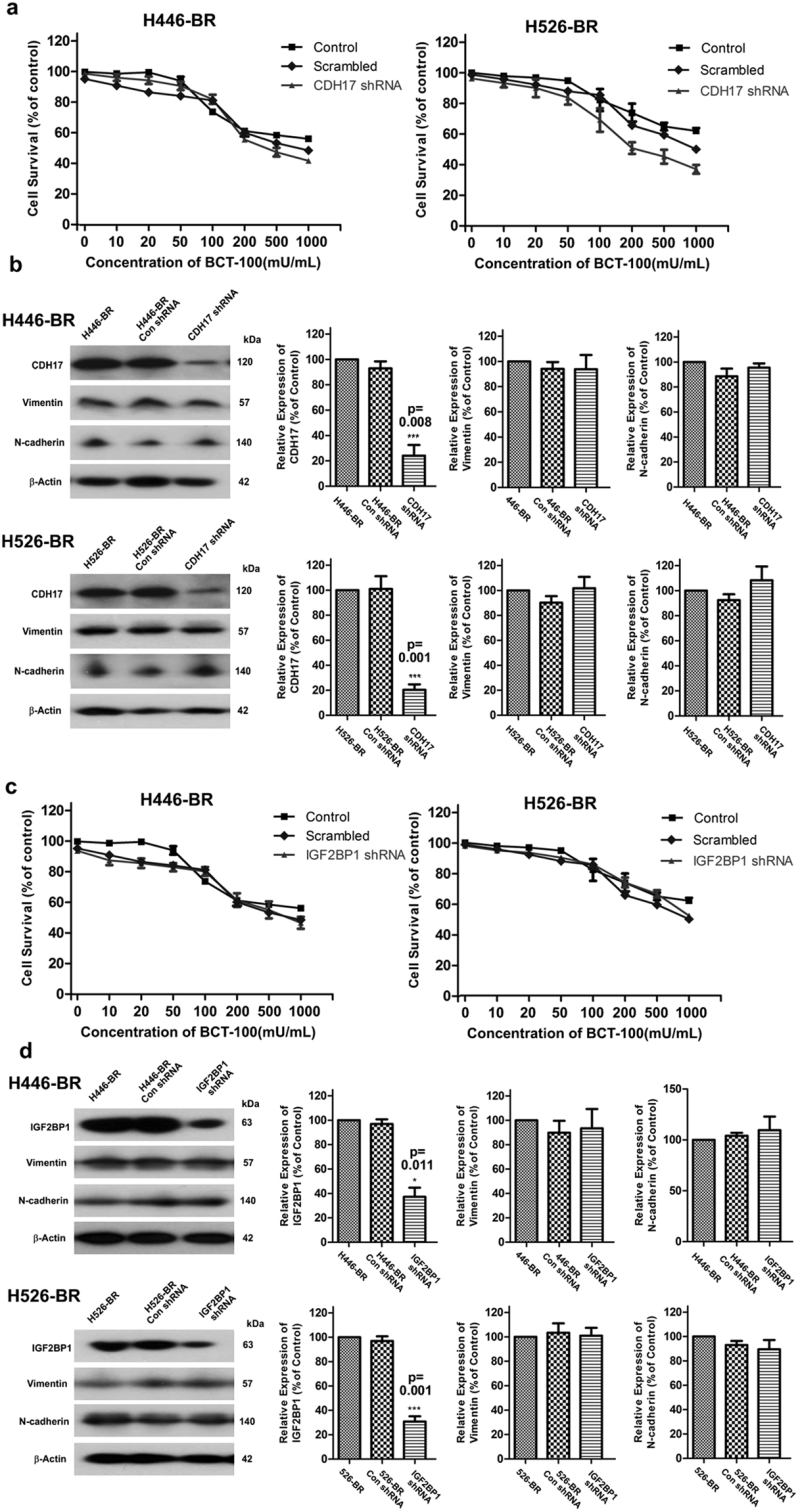
Knockdown of CDH17 and IGF2BP1 had very limited influence on BCT-100 resistance and EMT phenotype. (**a**) The cell survival was comparable in CDH17 silenced arm, scrambled and control group, and exposure time was 3 days. (**b**) CDH17 was downregulated while vimentin and N-cadherin were unaltered in the CDH17-silenced group. (**c**) The survival rate in the IGF2BP1 silenced arm, scrambled and control group. (**d**) IGF2BP1, vimentin and N-cadherin were tested by Western blot. *P < 0.05, **P < 0.01, ***P < 0.001.

In gene chip assay, CDH17 was up-regulated 39.39 and 12.81 fold while IGF2BP1 was increased 6.57 and 8.63 fold respectively in H446-BR and H526-BR cells. Both CDH17 and IGF2BP1 were functionally associated with cell proliferation and tumor metastasis. Nonetheless silencing CDH17 in BCT-100-resistant cell lines by specific RNA interference could not re-sensitize resistant cells to BCT-100 treatment (Fig. 4a), and EMT phenotype was unchanged (Fig. 4b). Similar phenomena were observed in IGF2BP1 silencing experiments (Fig. 4c,d).

### Silencing CNTN-1 enhanced sensitivity to BCT-100 treatment and attenuated EMT phenotype in resistant cell lines

**Figure 5:**
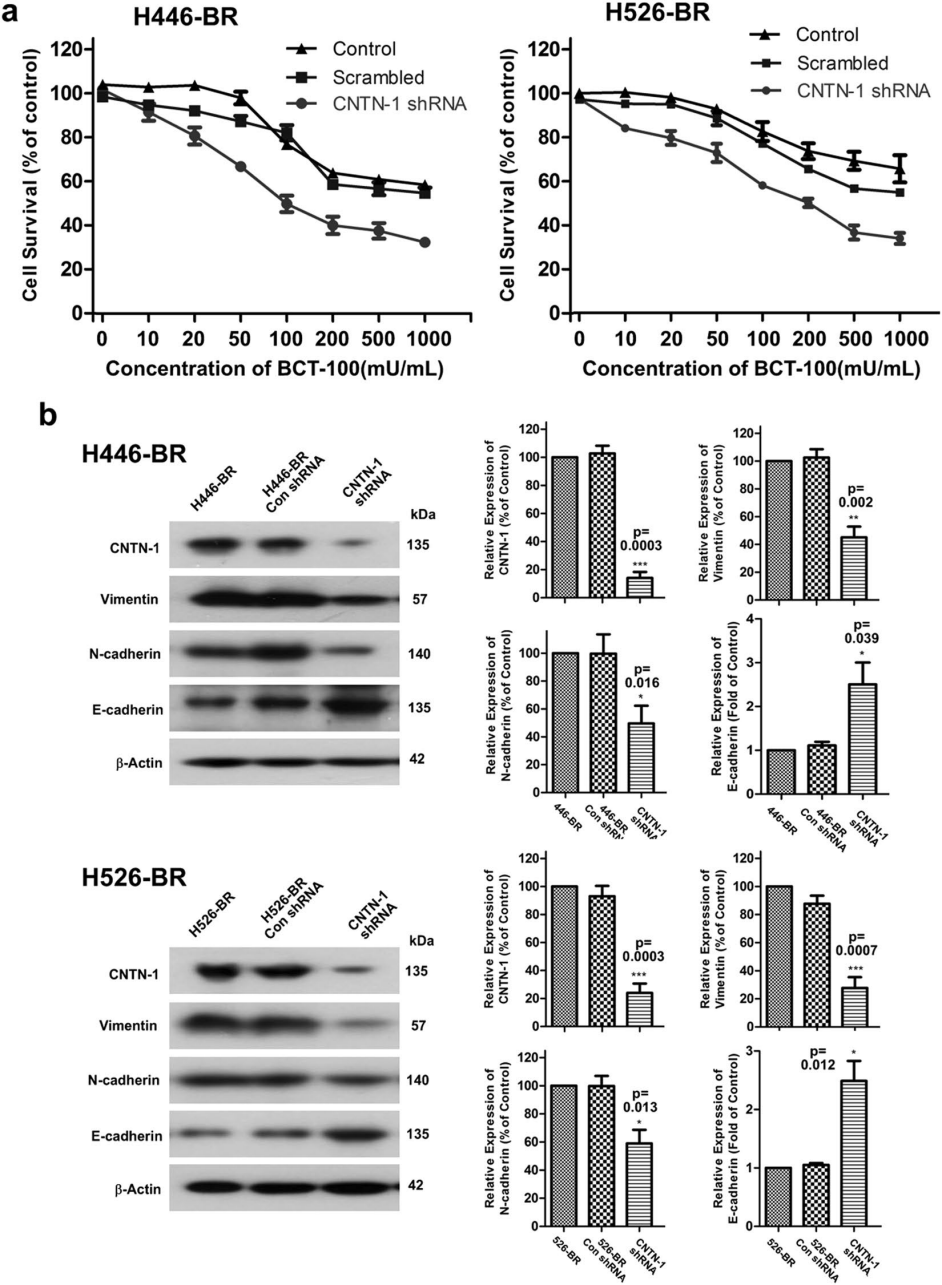
Silencing CNTN-1 enhanced sensitivity to BCT-100 treatment and attenuated the EMT phenotype in resistant cell lines. (**a**) MTT assay to test survival rate in CNTN-1 silenced arm, scrambled and control group, and treatment time was 3 days. (**b**) CNTN-1, vimentin, N-cadherin and E-cadherin were tested by Western blot.

MTT assay was performed to detect the survival rate following BCT-100 treatment after knockdown of CNTN-1 in H446-BR and H526-BR cell lines. The CNTN-1 silenced arms became more sensitive to BCT-100 than the control groups (Fig. 5a). The EMT-related proteins vimentin and N-cadherin were down-regulated, and E-cadherin was up-regulated in CNTN-1 silenced arms (Fig. 5b).

### CNTN-1 promoted EMT progression by activating the AKT pathway in resistant cells

**Figure 6:**
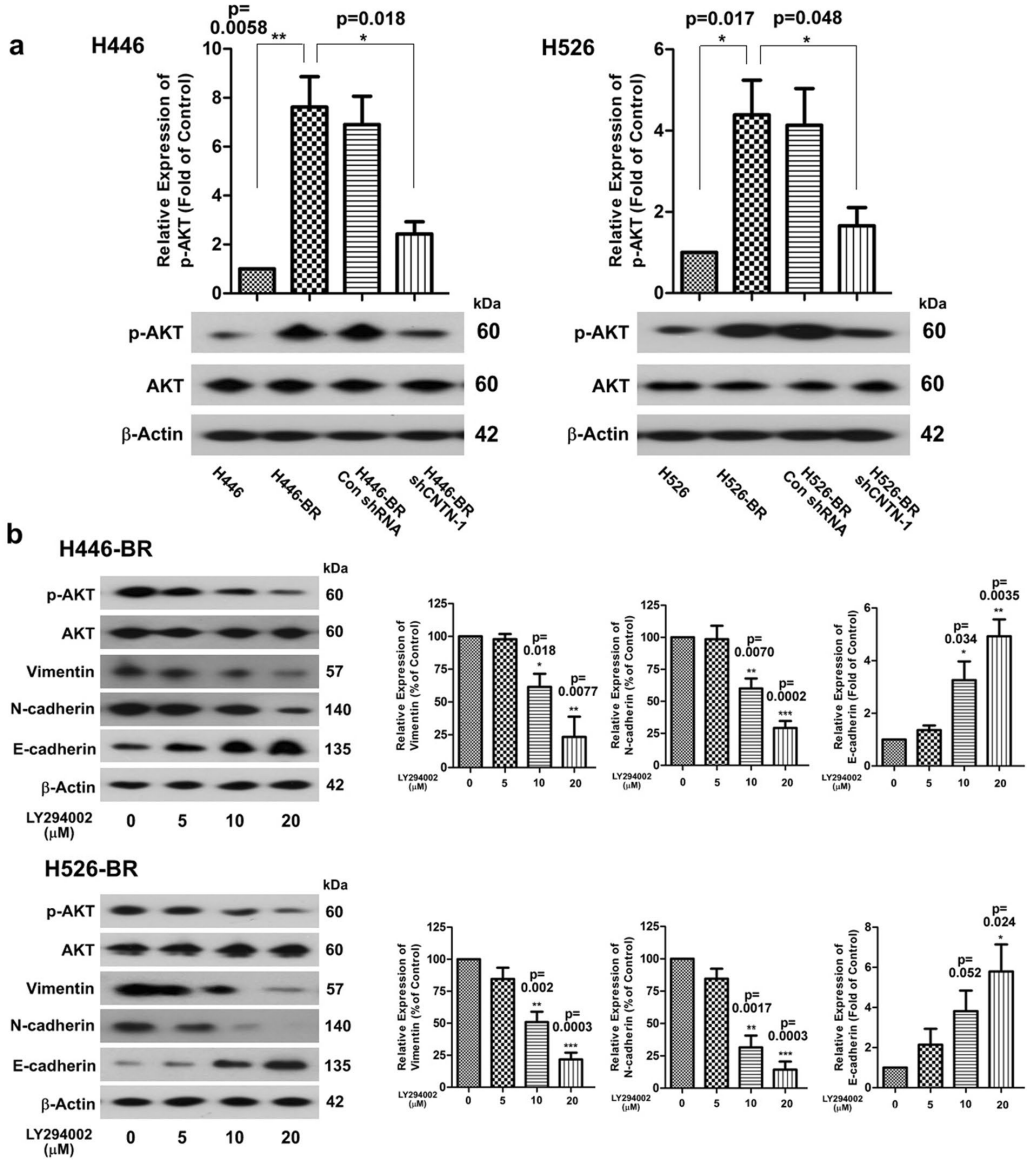
CNTN-1 promoted EMT progression via activation of the AKT pathway in resistant cells. (**a**) p-AKT was up-regulated in H446-BR and H526-BR cells, and down-regulated in the CNTN-1 silenced arms. (**b**) p-AKT, vimentin, N-cadherin and E-cadherin were tested by Western blot after LY294002 treatment (3 days).

The AKT signaling pathway plays a central role in cell survival and growth. To determine the role of AKT in BCT-100 resistance, we detected the basal expression of p-AKT in H446, H446-BR, H446-BR-shControl and H446-BR-shCNTN-1. The expression of p-AKT was up-regulated in H446-BR cells but down-regulated in the H446-BR-shCNTN-1 arms. Similar phenomena were observed in the H526 cell line (Fig. 6a). AKT inhibitor LY294002 was used to test the influence of p-AKT on the EMT phenotype in H446-BR and H526-BR cells. LY294002 inhibited the level of p-AKT in BCT-100-resistant cells accompanied by attenuation of EMT progression (vimentin and N-cadherin were decreased in a dose-dependent manner, and E-cadherin was up-regulated) (Fig. 6b).

## Discussion

BCT-100 has recently emerged as a potentially effective anti-cancer agent in arginine auxotrophic tumors including HCC^34^, melanoma^35^, malignant pleural mesothelioma^5^ and leukemia^6^. Although the underlying mechanisms of pegylated arginase in these cancer cells have been elucidated, drug resistance remains a major obstacle in the battle against cancer. To achieve a better understanding of the mechanism of BCT-100-based acquired drug resistance, we established two cell lines with BCT-100-acquired resistance (H446-BR and H526-BR) that exhibited different phenotypic features compared with parent cells (Supplementary Fig. S2). Resistant cells were more aggressive with higher migration ability, anoikis resistance and multidrug resistance to standard chemotherapeutic drugs. The EMT phenotype mediated by CNTN-1 via targeting of the AKT pathway was also accompanied by formation of resistant cells.

Accumulating evidence indicates a close relationship between EMT progression and tumor invasion and metastasis^36^. In addition, EMT has been cited as the means by which cancer cells gain migratory properties and leave the primary tumor site to distant metastases. In the tumor metastatic cascade, EMT exerts its function during the initial event when cancer cells with deficient epithelial characteristics leave the primary tumor site, invade adjacent normal tissue and eventually enter blood vessels to circulate to metastatic organs^11^. When completing metastasis, tumor cells are believed to undergo mesenchymal to epithelial transition (MET) to regain epithelial features and form secondary tumors^37^. During EMT progression, cancer cells are thought to obtain many cancer stem cell-like properties^38^. This is consistent with our findings (Oct-4 and nanog were up-regulated in H446-BR and H526-BR cells). Besides, increasing evidences have demonstrated stem cell-like cancer cells are regarded as the important contributor to multidrug resistance^39,40^. And EMT has been considered as a potential link between chronic obstructive pulmonary disease (COPD) and lung cancer^8,41^. Nonetheless tumor cells comprise various populations of cells with heterogeneous phenotypes. Interestingly, tumor cells that express both epithelial and mesenchymal biomarkers simultaneously can more effectively metastasize throughout the body and this is considered partial EMT^37^. Mesenchymal cancer cells are also thought to have a lower proliferation rate and among these cancer cells, some still have an undifferentiated metastatic ability for MET progression to a well-differentiated epithelial phenotype in distant organs^11^. Although EMT-MET homeostasis exists in tumor metastasis, the role of EMT has been frequently studied in cancer treatment. For example, EMT has been shown to be involved in paclitaxel resistance in epithelial ovarian carcinoma cells via an increase in mesenchymal markers and decreased epithelial adhesion molecule^42^.

CNTN-1, mapped to the chromosome 12q11-q12 region and composed of six C2 Ig-like repeats and four fibronectin type III (FNIII) domains, is the first identified member of the CNTN family of neural cell-recognition molecules^43,44^. CNTN-1 is an essential molecule in nervous system development and has been reported to be associated with metastasis, proliferation and drug resistance in gastric cancer^45^, thyroid cancer^46^, breast cancer^32^, HCC^47^ and lung adenocarcinoma^48^. In line with previous investigations, we observed that expression of CNTN-1 in resistant cells was higher than in progenitor cells, suggesting cancer stem cell-like characteristics with higher migration ability and anoikis resistance. It has also been demonstrated that silencing CNTN-1 renders resistant cells more sensitive to a chemotherapeutic drug than parent cells, and enhances the apoptosis induced by cisplatin, leading to inhibition of tumor invasion and metastasis in lung cancer^33,49^. It is notable that the level of CNTN1 in esophageal cancer cells was associated with vascular endothelial growth factor C (VEGF-C) that induced the recruitment of C/EBPα to interact with CNTN-1 promoter. Src kinase and p38 MAPK pathway were also involved in CNTN-1 up-regulation mediated by VEGF-C^50^. Moreover, the aggravated tumor invasion and migration in esophageal cancer cells have been attributed to increased expression of VEGF-C that can be reversed by a reduction in CNTN-1 level^51^.

In addition to preclinical study, CNTN-1 also indicates a poor prognosis in patients with advanced HCC. Compared with adjacent normal tissue, the expression of CNTN-1 in HCC tumors has been reported to be much higher, with CNTN-1 level positively correlated with tumor size, status of metastasis and TNM (tumor, node, metastasis) stage. The overall survival (OS) of HCC patients with CNTN-1 positive expression was significantly shorter than that of CNTN-1 negative patients (19.96 ± 9.39 VS 31.27 ± 11.45 months). Likewise, the disease-free survival (DFS) of a CNTN-1 positive group (13.61 ± 8.63 months) was much shorter than that of the negative group (24.51 ± 10.41 months)^47^. Additionally, the expression of CNTN-1 was positively correlated with lymphatic invasion in NSCLC patients who received cisplatin- or carboplatin-based therapy after surgery^49^. It is evident that CNTN-1 plays an essential role in tumor invasion and metastasis and may be a promising therapeutic target in cancer therapy.

It is well-known that the AKT signaling pathway plays a pivotal role in cell growth, metabolism, survival and apoptosis^52,53^. Generally, activation of this pathway contributes to pathogenesis of a variety of cancers, and many specific inhibitors have been approved by the FDA. For instance, MK-2206, an oral allosteric AKT inhibitor, can decrease cancer antigen and tumor size by approximately 60% and 23% respectively across different solid tumors^54^. Randomized controlled trials have shown that PI3K/AKT/mTOR inhibitors significantly improve the progression-free survival (PFS) of patients with advanced solid tumors (hazard ratio (HR) = 0.79; 95% confidence intervals (CI): 0.71–0.88)^55^. Further, the AKT signaling pathway underlies drug resistance in many cancers such as lung cancer^56^, HCC^57^ and melanoma^58^. Similarly, activation of the AKT pathway also contributes to arginine deiminase (ADI) resistance in melanoma cells^59^. The AKT pathway may be a potential target in drug resistance mechanisms.

CNTN-1 belongs to the cell adhesion molecules (CAMs), which are composed of the immunoglobulin super family of cell adhesion molecules (IgCAMs), cadherins, integrins, and the superfamily of C-type of lectin-like domains proteins (CTLDs). Theoretically, CNTN-1 has interaction with cadherins to regulate biological function. In fact, it has been demonstrated that CNTN-1 reduced the expression of E-cadherin through AKT pathway in lung cancer^60^. Therefore, CNTN-1 might be a crucial factor in EMT progression.

In our study, knockdown of CNTN-1 re-sensitized H446-BR and H526-BR cells to BCT-100 treatment and was accompanied by attenuation of EMT progression. The AKT signaling pathway was activated in resistant cells and AKT inhibitor LY294002 reversed EMT progression in BCT-100 resistant cells. These results indicate that the AKT pathway was involved in EMT progression.

Growing evidence indicates that CDH17 and IGF2BP1 are related to cell proliferation and tumor metastasis^26,61^. These two molecules were considered potential targets in our study design since they were highly expressed in BCT-100 resistant cells. Nonetheless silencing these two genes did not affect sensitivity to BCT-100 treatment or EMT phenotype, suggesting they might not be essential to BCT-100 acquired resistance in SCLC.

There were limitations in our study. Although we obtained two BCT-100-acquired resistance SCLC cell lines, the number was still limited. It would be important to expand the number of cell lines in future study to confirm that our findings are universal, not cell line specific. Besides, LY294002 is generally considered as a pan PI3K-AKT inhibitor and has been shown to block PI3K-AKT phosphorylation and kinase activity. But PI3K-AKT signaling pathway has many biological functions. It would be more convincing if we select specific AKT inhibitor such as MK-2206 in the future. The BCT-100 resistant cells used in this study may differ to those in clinically resistant patient samples. It would be useful to include CNTN-1 expression in clinical central airway samples and SCLC samples in future exploration. In summary, the BCT-100 resistant cells and parent cells have biologically distinct phenotypes. H446-BR and H526-BR cells exhibited more aggressive migration, and CNTN-1 promoted BCT-100 resistance in these two cell lines through induction of EMT progression by activating AKT pathways.

## Methods

### Chemicals and reagents

BCT-100 was provided by Bio-cancer Treatment International Limited, Hong Kong. Cell culture medium (RPMI-1640) and fetal bovine serum (FBS) were purchased from Thermo Fisher Scientific (Carlsbad, CA, USA). Antibodies including E-cadherin, N-cadherin, vimentin, CDH17, CNTN-1, IGF2BP1, p-AKT, AKT and β-Actin were purchased from Cell Signaling Technology (Danvers, MA, USA). Cisplatin and etoposide were bought from Cayman Chemical (Ann Arbor, MI, USA).

### Cell lines and culture

Two SCLC cell lines (H446 (NCI-H446 (ATCC® HTB-171™) and H526 (NCI-H526 [H526] (ATCC® CRL-5811™)) were obtained from the American Type Culture Collection (Manassas, VA, USA). The cell lines were not tested for mycoplasma or cell authentication. Both H446 (typical SCLC) and H526 (variant SCLC) cell lines were relatively sensitive to BCT-100^62^. They were both cultured in RPMI-1640 medium enriched with 10% FBS in a CO_2_ incubator (Thermo Fisher). The incubator was set at a humidified environment with 5% CO_2_ at 37 °C.

### Establishment of a BCT-100-resistant cell line

H446 and H526 cells were exposed to increasing concentrations of BCT-100 for at least six months. The durations of exposure for parent cells to different concentrations were different: around 2 weeks for lower concentrations (5, 10 and 20 mU/mL) and 3–4 weeks for higher concentrations (50, 100 and 200 mU/mL). The dosage of BCT-100 was elevated when around 20% cells death was observed at that particular concentration. The timing of exposure was similar in establishing the resistant H446-BR and H526-BR cell lines. Cells were maintained for 2 months in medium that contained 200 mU/mL of BCT-100 for subsequent experiments. The cell lines were not tested for cell authentication.

### Cell proliferation assay

Briefly, cells (10^4^ per well) were seeded in a 96-well plate. After exposure to drug, 10 μL of MTT (bromide 3-(4,5-dimethylthiazol-2-yl) -2,5-diphenyltetrazolium) solution (0.5 mg/mL) (Sigma–Aldrich) was loaded into each well for incubation (3 hours), followed by the addition of 100 μL triple-lysis solution (5% isobutanol, 10% sodium dodecyl sulfate, 0.012 mol/L HCl in water) for 2 hours. The microplate reader Fluo Star Optima (Bmg Labtec GmbH, Ortenberg, Germany) was used to test the absorbance value at 570 nm after crystals were dissolved.

### Wound-healing assay

Cells (5 × 10^5^ per well) were seeded and cultured into 6-well plates with complete medium. The wound healing rated measured was the combination effect of migration and enhanced cell proliferation. A 200- μL pipette tip was used to scratch a straight line in the middle of the well when cells were approximately 80% confluent. Cells were washed with PBS and medium refreshed after taking images at different time points using a microscope (Nikon SMZ745, Nikon, Japan).

### Anoikis resistance assay

In brief, cells were seeded into a 6-well plate that was coated with poly-2-hydroxyethyl methacrylate (12 mg/mL, Sigma-Aldrich). Cell clones were captured by microscope (Nikon SMZ745, Nikon, Japan) and the apoptotic rate examined by flow cytometry employing the annexin V-PE/7-AAD kit. Apoptotic rate was measured according to the manufacturer’s instructions. A Beckman Coulter FC500 flow cytometer (Beckman Coulter Inc., Brea, CA, USA) was used to analyze the stained cells.

### RNA isolation and real-time polymerase chain reaction (RT-qPCR) analysis

TRIzol reagent (Invitrogen, USA) was used to extract RNA from harvested samples. In brief, samples were resuspended in TRIzol reagent and chloroform in sequence for extraction. The mixture was incubated at room temperature for 15 minutes and centrifuged at 12,000 g for 15 minutes at 4 °C. The upper colorless aqueous phase was transferred to a RNase-free tube and 200 μL of isopropanol added. The mixture was incubated for 10 minutes at room temperature and centrifuged at 12,000 g for 15 minutes at 4 °C. The pellets were saved as RNA and re-dissolved in ddH_2_O. Reverse transcription of RNA was performed using a High Capacity cDNA Reverse Transcription kit (Applied Biosystems, Lithuania) according to the manufacturer’s instructions. Quantitative RT-PCR assay was performed with SYBR Green Real-Time PCR Master Mixes (Thermo Fisher Scientific, USA) based on the manufacturer’s protocol. The calculation was based on the classical method established by Livak and Schmittgen^63^. The forward and reverse primers tested were as follows:

CDH17-F-5′-GCCAATCCTCCTGCTGTG-3′,

CDH17-R-5′-GCAACCTGGAGATTGTGAGT-3′

CNTN-1-F-5′-GCCCATGACAAAGAAGAAGC-3′,

CNTN-1-R-5′-CGACATGATCCCAGGTGATT-3′,

IGF2BP1-F-5′-CAGGAGATGGTGCAGGTGTTTATCC-3′,

IGF2BP1-R-5′-GTTTGCCATAGATTCTTCCCTGAGC-3′,

GAPDH-F-5′-AGCCACATCGCTCAGACACC-3′,

GAPDH-R-5′-GTACTCAGCGCCAGCATCG-3′.

### Short hairpin RNA (shRNA) transfection

Briefly, 5 × 10^5^ cells per well were seeded in a 6-well plate. Cells were incubated with polybrene prior to the addition of lentiviral particles followed by incubation for 24 hours. Complete medium was added for a further 48 hours incubation. Puromycin dihydrochloride was used to select the stable silenced cells.

### Western blotting

Total protein was extracted with RIPA buffer (1 mM EDTA, 10 mM Tris-buffer (pH 7.6), 10 mM KCl, 1.5 mM MgCl_2_, 1 mM phenylmethylsulphonyl fluoride (PMSF)) supplemented with protease inhibitor for 1 hour. Proteins (40 µg) were subjected to sodium dodecyl sulfate polyacrylamide gel electrophoresis (SDS–PAGE) (7.5–15%) and transferred onto nitrocellulose membranes (GE Healthcare, Buckinghamshire, UK). The membranes were blocked for 1 hour at room temperature with 5% non-fat dry milk or 3% BSA in TBST (tris-buffered saline (pH 7.4), 0.1% Tween-20). Membranes were incubated with specific monoclonal or polyclonal primary antibodies at 4 °C overnight. Membranes were washed three times with TBST, 10 minutes per wash. Subsequently, membranes were incubated with corresponding horseradish peroxidase (HRP) secondary antibodies (Cell Signaling Technology, MA, USA) for 1 hour at room temperature and then washed for 10 minutes with TBST buffer three times. Signal detection was performed using an enhanced chemiluminescence (ECL) kit (GE Healthcare) and quantified using GelQuantNET software (Biochem Lab Solutions, CA, USA). Details of primary antibodies used in this study are shown in Supplementary Table SII.

### Immunofluorescence staining

Cells were crawling on slides followed by fixation and permeabilization. Samples were blocked with 3% BSA for 1 hour at room temperature, followed by incubation with E-cadherin (Cell Signaling Technology, Danvers, MA, USA) antibody overnight at 4 °C. After washing with PBST for 30 minutes, Alexa Fluor anti-rabbit (Life Technologies) antibody was applied followed by incubation for 1 hour protected from light. The slides were mounted with Prolong Gold antifade reagent with DAPI (Life Technologies). Pictures were obtained using a Zeiss fluorescence microscope.

### Gene chip assay

Total RNAs extracted from H446, H446-BR, H526 and H526-BR cells was submitted to the Centre for Genomic Sciences (HKU) for Quality Control testing. Acceptable samples were subjected to reverse transcription and hybridization using Affymetrix Human Transcriptome Array (HTA) 2.0. The difference of RNA integrity number (RIN) and ribosomal RNA ratio in each sample should be minor. Each arm was conducted with three samples. Data were analyzed using Partek Genomics Suite software (Partek Inc, MI, USA) according to the manufacturer’s instructions.

### Statistical analysis

All data are obtained by three independent assays and presented as mean ± standard deviation (SD), with between-group differences analyzed by two-tailed Student’s t-test. The protein expression levels (by Western blots) of treatment arms were individually normalized with controls in separate experiments. A p-value < 0.05 was considered statistically significant (*P < 0.05, **P < 0.01, ***P < 0.001). All statistical analyses were performed using Prism 5 (GraphPad Software, La Jolla, CA, USA). The figure processing manipulations were conducted by MacromediaFireworks8 (Adobe, CA, USA).

## Supplementary information

Supplementary Info File revised

## Data Availability

The datasets used during and/or analysed the present study are available from the corresponding author upon reasonable request.

## References

[1] Oronsky B, Reid TR, Oronsky A, Carter CA (2017). What’s New in SCLC? A Review. Neoplasia.

[2] De Santo C (2018). The arginine metabolome in acute lymphoblastic leukemia can be targeted by the pegylated-recombinant arginase I BCT-100. Int J Cancer.

[3] Chow AK (2012). Anti-tumor efficacy of a recombinant human arginase in human hepatocellular carcinoma. Curr Cancer Drug Targets.

[4] Lam TL (2011). Recombinant human arginase inhibits the *in vitro* and *in vivo* proliferation of human melanoma by inducing cell cycle arrest and apoptosis. Pigment Cell Melanoma Res.

[5] Lam SK (2017). Growth suppressive effect of pegylated arginase in malignant pleural mesothelioma xenografts. Respir Res.

[6] Mussai F (2015). Arginine dependence of acute myeloid leukemia blast proliferation: a novel therapeutic target. Blood.

[7] Xu S, Lam SK, Cheng PN, Ho JC (2018). Recombinant human arginase induces apoptosis through oxidative stress and cell cycle arrest in small cell lung cancer. Cancer Sci.

[8] Eapen MS (2018). Chronic Obstructive Pulmonary Disease and Lung Cancer: Underlying Pathophysiology and New Therapeutic Modalities. Drugs.

[9] Smith, B. N. & Bhowmick, N. A. Role of EMT in Metastasis and Therapy Resistance. *J Clin Med* **5** (2016). 10.3390/jcm5020017 PMC4773773 26828526

[10] Cai M (2015). Adam17, a Target of Mir-326, Promotes Emt-Induced Cells Invasion in Lung Adenocarcinoma. Cell Physiol Biochem.

[11] Bill R, Christofori G (2015). The relevance of EMT in breast cancer metastasis: Correlation or causality?. FEBS Lett.

[12] Shang Y, Cai X, Fan D (2013). Roles of epithelial-mesenchymal transition in cancer drug resistance. Curr Cancer Drug Targets.

[13] Mitra A, Mishra L, Li S (2015). EMT, CTCs and CSCs in tumor relapse and drug-resistance. Oncotarget.

[14] Voulgari A, Pintzas A (2009). Epithelial-mesenchymal transition in cancer metastasis: mechanisms, markers and strategies to overcome drug resistance in the clinic. Biochim Biophys Acta.

[15] Ma JL, Zeng S, Zhang Y, Deng GL, Shen H (2016). Epithelial-mesenchymal transition plays a critical role in drug resistance of hepatocellular carcinoma cells to oxaliplatin. Tumour Biol.

[16] Ma D (2018). Paclitaxel increases the sensitivity of lung cancer cells to lobaplatin via PI3K/Akt pathway. Oncol Lett.

[17] Yun M (2015). Cinnamaldehyde derivative (CB-PIC) sensitizes chemo-resistant cancer cells to drug-induced apoptosis via suppression of MDR1 and its upstream STAT3 and AKT signalling. Cell Physiol Biochem.

[18] Deying, W. *et al*. CAF-derived HGF promotes cell proliferation and drug resistance by up-regulating the c-Met/PI3K/Akt and GRP78 signalling in ovarian cancer cells. *Biosci Rep* **37** (2017). 10.1042/BSR20160470 PMC5469328 28258248

[19] Chen JR (2016). Timosaponin A-III reverses multi-drug resistance in human chronic myelogenous leukemia K562/ADM cells via downregulation of MDR1 and MRP1 expression by inhibiting PI3K/Akt signaling pathway. Int J Oncol.

[20] Meng W (2015). Correlation of cadherin-17 protein expression with clinicopathological features and prognosis of patients with sporadic gastric cancer. Brazilian journal of medical and biological research = Revista brasileira de pesquisas medicas e biologicas.

[21] Wang J (2013). Cadherin-17 induces tumorigenesis and lymphatic metastasis in gastric cancer through activation of NFkappaB signaling pathway. Cancer biology & therapy.

[22] Lee NP, Poon RT, Shek FH, Ng IO, Luk JM (2010). Role of cadherin-17 in oncogenesis and potential therapeutic implications in hepatocellular carcinoma. Biochimica et biophysica acta.

[23] Bartolome RA (2014). Cadherin-17 interacts with alpha2beta1 integrin to regulate cell proliferation and adhesion in colorectal cancer cells causing liver metastasis. Oncogene.

[24] Bell JL (2013). Insulin-like growth factor 2 mRNA-binding proteins (IGF2BPs): post-transcriptional drivers of cancer progression?. Cellular and molecular life sciences: CMLS.

[25] Gutschner T (2014). Insulin-like growth factor 2 mRNA-binding protein 1 (IGF2BP1) is an important protumorigenic factor in hepatocellular carcinoma. Hepatology.

[26] Faye MD (2015). IGF2BP1 controls cell death and drug resistance in rhabdomyosarcomas by regulating translation of cIAP1. Oncogene.

[27] Su Y (2016). MicroRNA-140-5p targets insulin like growth factor 2 mRNA binding protein 1 (IGF2BP1) to suppress cervical cancer growth and metastasis. Oncotarget.

[28] Bizzoca A, Corsi P, Gennarini G (2009). The mouse F3/contactin glycoprotein: structural features, functional properties and developmental significance of its regulated expression. Cell Adh Migr.

[29] Colakoglu G, Bergstrom-Tyrberg U, Berglund EO, Ranscht B (2014). Contactin-1 regulates myelination and nodal/paranodal domain organization in the central nervous system. Proc Natl Acad Sci U S A.

[30] Tan Q (2016). Genomic Alteration During Metastasis of Lung Adenocarcinoma. Cell Physiol Biochem.

[31] Chen DH, Yu JW, Jiang BJ (2015). Contactin 1: A potential therapeutic target and biomarker in gastric cancer. World J Gastroenterol.

[32] Chen N (2018). Overexpression of Contactin 1 promotes growth, migration and invasion in Hs578T breast cancer cells. BMC Cell Biol.

[33] Su JL (2006). Knockdown of contactin-1 expression suppresses invasion and metastasis of lung adenocarcinoma. Cancer Res.

[34] Cheng PN (2007). Pegylated recombinant human arginase (rhArg-peg5,000mw) inhibits the *in vitro* and *in vivo* proliferation of human hepatocellular carcinoma through arginine depletion. Cancer Res.

[35] De Santo C (2018). Metabolic therapy with PEG-arginase induces a sustained complete remission in immunotherapy-resistant melanoma. J Hematol Oncol.

[36] Yoshida T (2014). Eribulin mesilate suppresses experimental metastasis of breast cancer cells by reversing phenotype from epithelial-mesenchymal transition (EMT) to mesenchymal-epithelial transition (MET) states. Br J Cancer.

[37] Saitoh, M. Involvement of partial EMT in cancer progression. *J Biochem* (2018). 10.1093/jb/mvy047 29726955

[38] Nieto MA (2013). Epithelial plasticity: a common theme in embryonic and cancer cells. Science.

[39] Ma J (2016). Reversing drug resistance of soft tumor-repopulating cells by tumor cell-derived chemotherapeutic microparticles. Cell Res.

[40] Butler SJ, Richardson L, Farias N, Morrison J, Coomber BL (2017). Characterization of cancer stem cell drug resistance in the human colorectal cancer cell lines HCT116 and SW480. Biochem Biophys Res Commun.

[41] Sohal SS (2015). Chronic Obstructive Pulmonary Disease (COPD) and Lung Cancer: Epithelial Mesenchymal Transition (EMT), the Missing Link?. EbioMedicine.

[42] Kajiyama H (2007). Chemoresistance to paclitaxel induces epithelial-mesenchymal transition and enhances metastatic potential for epithelial ovarian carcinoma cells. Int J Oncol.

[43] Shimoda Y, Watanabe K (2009). Contactins: emerging key roles in the development and function of the nervous system. Cell Adh Migr.

[44] Berglund EO, Ranscht B (1994). Molecular cloning and *in situ* localization of the human contactin gene (CNTN1) on chromosome 12q11-q12. Genomics.

[45] Chen DH, Yu JW, Wu JG, Wang SL, Jiang BJ (2015). Significances of contactin-1 expression in human gastric cancer and knockdown of contactin-1 expression inhibits invasion and metastasis of MKN45 gastric cancer cells. J Cancer Res Clin Oncol.

[46] Shi K (2015). Contactin 1 as a potential biomarker promotes cell proliferation and invasion in thyroid cancer. Int J Clin Exp Pathol.

[47] Li GY, Huang M, Pan TT, Jia WD (2016). Expression and prognostic significance of contactin 1 in human hepatocellular carcinoma. Onco Targets Ther.

[48] Zhang R (2017). CNTN-1 Enhances Chemoresistance in Human Lung Adenocarcinoma Through Induction of Epithelial-Mesenchymal Transition by Targeting the PI3K/Akt Pathway. Cell Physiol Biochem.

[49] Zhang R (2015). Increased sensitivity of human lung adenocarcinoma cells to cisplatin associated with downregulated contactin-1. Biomed Pharmacother.

[50] Su JL (2006). The VEGF-C/Flt-4 axis promotes invasion and metastasis of cancer cells. Cancer Cell.

[51] Liu P (2011). VEGF-C promotes the development of esophageal cancer via regulating CNTN-1 expression. Cytokine.

[52] Meric-Bernstam F (2012). PIK3CA/PTEN mutations and Akt activation as markers of sensitivity to allosteric mTOR inhibitors. Clin Cancer Res.

[53] Hassan B, Akcakanat A, Holder AM, Meric-Bernstam F (2013). Targeting the PI3-kinase/Akt/mTOR signaling pathway. Surg Oncol Clin N Am.

[54] Yap TA (2011). First-in-man clinical trial of the oral pan-AKT inhibitor MK-2206 in patients with advanced solid tumors. J Clin Oncol.

[55] Li X (2018). Efficacy of PI3K/AKT/mTOR pathway inhibitors for the treatment of advanced solid cancers: A literature-based meta-analysis of 46 randomised control trials. PLoS One.

[56] Phuchareon J, McCormick F, Eisele DW, Tetsu O (2015). EGFR inhibition evokes innate drug resistance in lung cancer cells by preventing Akt activity and thus inactivating Ets-1 function. Proc Natl Acad Sci U S.

[57] Zhang H, Wang Q, Liu J, Cao H (2018). Inhibition of the PI3K/Akt signaling pathway reverses sorafenib-derived chemo-resistance in hepatocellular carcinoma. Oncol Lett.

[58] Chi M, Ye Y, Zhang XD, Chen J (2014). Insulin induces drug resistance in melanoma through activation of the PI3K/Akt pathway. Drug Des Devel Ther.

[59] Tsai WB (2012). Activation of Ras/PI3K/ERK pathway induces c-Myc stabilization to upregulate argininosuccinate synthetase, leading to arginine deiminase resistance in melanoma cells. Cancer Res.

[60] Yan J, Wong N, Hung C, Chen WX, Tang D (2013). Contactin-1 reduces E-cadherin expression via activating AKT in lung cancer. PLoS One.

[61] Li R, Yang HQ, Xi HL, Feng S, Qin RH (2017). Inhibition of CDH17 gene expression via RNA interference reduces proliferation and apoptosis of human MKN28 gastric cancer cells. Int J Oncol.

[62] Xu, S., Lam, S. K., Cheng, P. N. & Ho, J. C. Recombinant human arginase induces apoptosis via oxidative stress and cell cycle arrest in small cell lung cancer. *Cancer Sci* (2018). 10.1111/cas.13782 PMC6215893 30155941

[63] Livak KJ, Schmittgen TD (2001). Analysis of relative gene expression data using real-time quantitative PCR and the 2(-Delta Delta C(T)) Method. Methods.

