# Supplementary material for: Contactin 1 modulates pegylated arginase resistance in small cell lung cancer through induction of epithelial–mesenchymal transition
Source: Sci Rep. 2019 Aug 19;9:12030. doi: 10.1038/s41598-019-48476-8 (PMC6700313; doi:10.1038/s41598-019-48476-8)
Supplement: Supplementary file 1 — Supplementary Info File revised [file 41598_2019_48476_MOESM1_ESM.pdf]

Contactin 1 modulates pegylated arginase resistance in small cell lung cancer through induction of epithelial–mesenchymal transition

Shi Xu[1], Sze-Kwan Lam[1], Paul Ning-Man Cheng[2] and James Chung-Man Ho[1*]

[1]Division of Respiratory Medicine, Department of Medicine, The University of Hong Kong, Queen Mary Hospital, Pokfulam, Hong Kong SAR, China

[2]Bio-cancer Treatment International, 511-513, Bioinformatics Building, Hong Kong Science Park, Tai Po, Hong Kong SAR, China

Address for correspondence:

Dr. James C. Ho MD, FRCP

Department of Medicine, The University of Hong Kong

Queen Mary Hospital, Pokfulam, Hong Kong SAR, China

Tel: (852) 2255 4999 Fax: (852) 2872 5828 Email: jhocm@hku.hk

Supplementary Figure 1

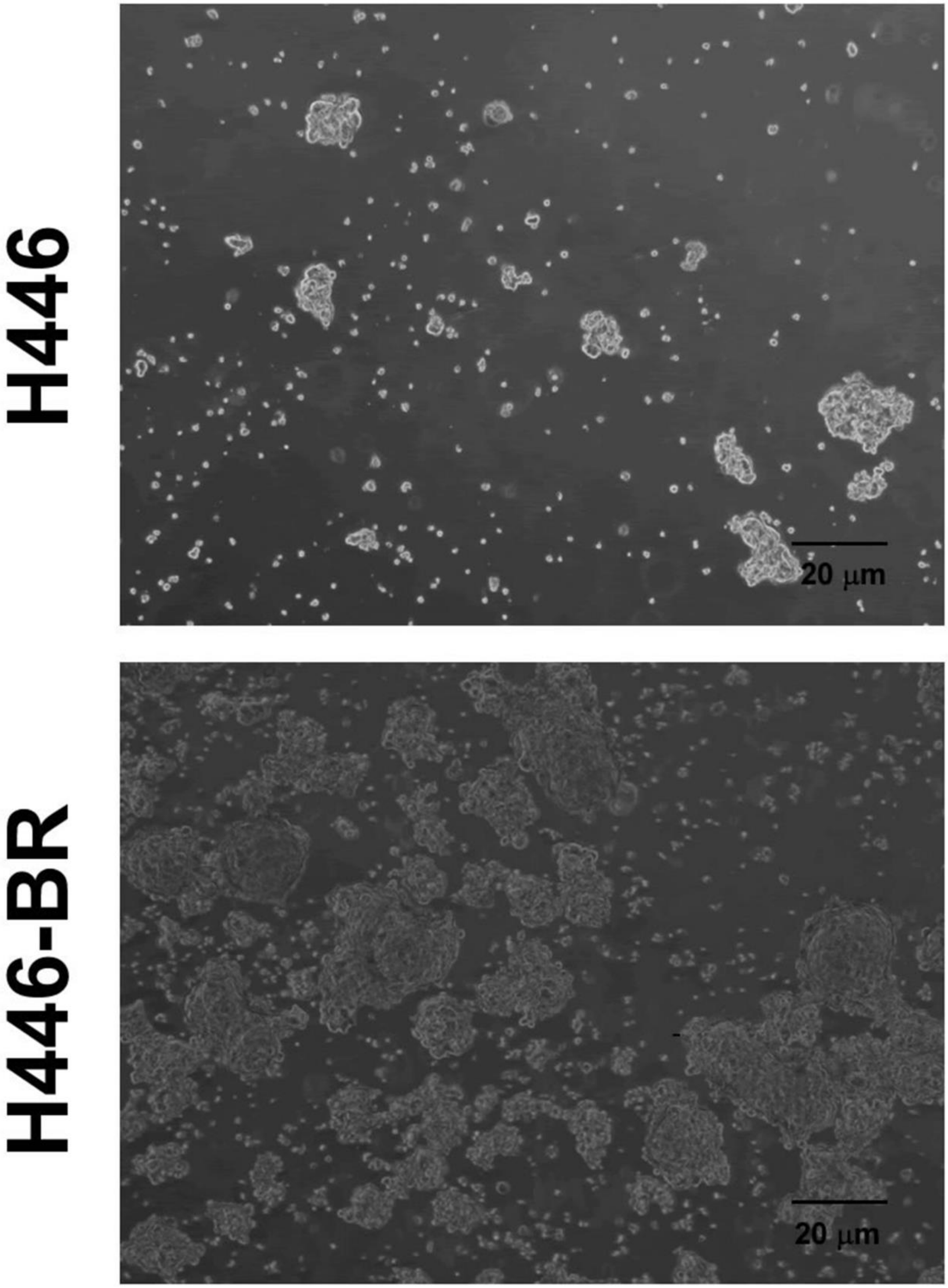

Supplementary Figure 2

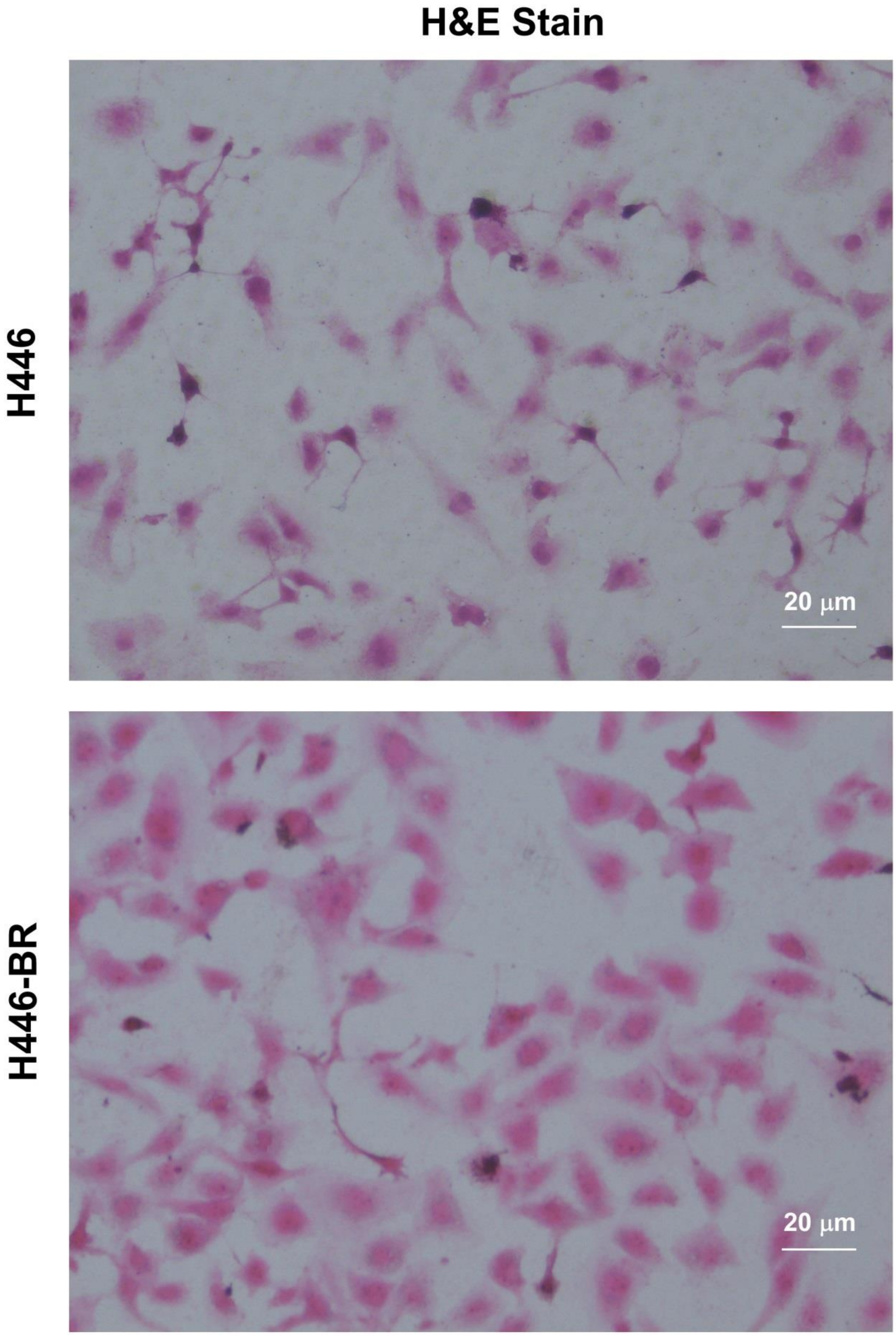

Supplementary table SI Gene alteration over 3 folds in both H446-BR and H526-BR cells

Gene symbol	Fold change in H446-BR	Fold change in H526-BR	Gene function
C11orf87	-26.8795	13.365	Unknown
CDH17	39.3945	12.8154	Metastasis
IGF2BP1	6.57612	8.63729	Proliferation, metastasis
PIK3CG	-5.24338	8.38477	Modulator of extracellular signals
RAB27B	5.20434	8.12985	Myocardial injuries
ARID5B	11.5541	5.76759	Acute lymphoblastic leukemia
LRRC31	-7.81027	5.67396	Esophageal epithelium
SCIN	-14.0771	-6.55804	Cortical actin
SLC17A8	-6.9557	-7.19254	Vesicular glutamate transporter
NUGGC	-16.6545	-7.26551	Nuclear GTPase
AKR1C4	-5.54643	3.74524	Aldo-keto reductase
BACE2	-4.03104	-6.16791	Amyloid beta generation, AD
CLIC5	-5.17188	3.09714	Actin cytoskeleton of placental microvilli
EYA1	23.6924	3.29794	Kidney, branchial arches, eye, and ear development
CNTN1	4.63378	16.8903	Cell adhesion molecule
GALNT14	3.6161	-3.67249	Chemotherapy responses
GNAT3	-4.72415	8.40745	Basic taste
HIST1H1T	-3.53835	-3.62821	DNA linker
HIST1H2AB	-6.9557	-7.19254	DNA linker
HIST1H4H	3.50764	3.76697	DNA linker
KLHL14	-6.69832	-4.1507	Unknown
KRT40	-6.69832	-4.65409	Keratin gene
LIN7A	11.2129	3.07636	Synaptic regulation
LINC00571	3.13601	3.57143	Non-protein coding
LOC283454	4.37566	3.18046	Unknown
LPCAT2	4.93715	8.64026	Phosphatidylcholine generation
MAGEC1	7.73534	4.6839	Cancer testis antigen
MAOA	5.49936	4.72268	Catalyze oxidative deamination
MUC13	-11.4882	4.74639	Form gels
PCAT18	-3.71491	-7.92082	Prostate cancer associated transcript
PHYHIPL	3.92407	5.05792	Tumor suppressor in gastric carcinoma
RGS5	-9.40102	3.53979	Heterotrimeric G proteins regulation
SCN1A	-4.10093	-3.57921	Sodium channels
SESN3	3.12125	3.10213	Reduce ROS
STK32A	11.1959	3.52189	Serine/threonine kinase 32a
SUCNR1	-6.67542	3.45295	Retinal angiogenesis
TOX3	3.68202	3.20861	Modify chromatin structure
ZDHHC15	9.93099	3.21904	Anchor proteins to cell membrane

Supplementary table SII List of primary antibodies used in this study

Name	Species	Manufacture	Cat#	Moclecular weight (kDa)	Dilution factor	Supplier
Vimentin	Rabbit	Cell Signaling Technology	5741	57	1:1000	Gene Company Limited
N-Cadherin	Rabbit	Cell Signaling Technology	4061	140	1:1000	Gene Company Limited
E-Cadherin	Rabbit	Cell Signaling Technology	3195	135	1:1000	Gene Company Limited
OCT-4	Rabbit	Cell Signaling Technology	2750	45	1:1000	Gene Company Limited
β-Actin	Mouse	Sigma-Aldrich	A1978	42	1:5000	Tin Hang Technology limited
Nanog	Rabbit	Cell Signaling Technology	4903	42	1:1000	Gene Company Limited
CDH17	Rabbit	Cell Signaling Technology	42919	120	1:1000	Gene Company Limited
IGF2BP1	Mouse	Santa Cruz Biotechnology	SC-166344	63	1:200	Genetimes Technology International Holding Limited
CNTN-1	Mouse	Santa Cruz Biotechnology	SC-136133	135	1:200	Genetimes Technology International Holding Limited
p-AKT	Rabbit	Cell Signaling Technology	4060	60	1:1000	Gene Company Limited
AKT	Rabbit	Cell Signaling Technology	9272	60	1:1000	Gene Company Limited
